# Anticancer Activities of Ginsenosides, the Main Active Components of Ginseng

**DOI:** 10.1155/2021/8858006

**Published:** 2021-02-03

**Authors:** Heeok Hong, Delgerzul Baatar, Seong Gu Hwang

**Affiliations:** ^1^Department of Animal Science and Technology, Konkuk University, Seoul 05029, Republic of Korea; ^2^Laboratory of Genetics, Institute of Biology, Mongolian Academy of Sciences, Peace Avenue 13330, Ulaanbaatar, Mongolia; ^3^Department of Animal Life and Environmental Science, Hankyong National University, Anseong City 17579, Republic of Korea

## Abstract

Cancer incidence rate has been increasing drastically in recent years. One of the many cancer treatment methods is chemotherapy. Traditional medicine, in the form of complementary and alternative therapy, is actively used to treat cancer, and many herbs and active ingredients of such therapies are being intensely studied to integrate them into modern medicine. Ginseng is traditionally used as a nourishing tonic and for treating various diseases in Asian countries. The therapeutic potential of ginseng in modern medicine has been studied extensively; the main bioactive component of ginseng is ginsenosides, which have gathered attention, particularly for their prospects in the treatment of fatal diseases such as cancer. Ginsenosides displayed their anticancer and antimetastatic properties not only via restricting cancer cell proliferation, viability, invasion, and migration but also by promoting apoptosis, cell cycle arrest, and autophagy in several cancers, such as breast, brain, liver, gastric, and lung cancer. Additionally, ginsenosides can work synergistically with already existing cancer therapies. Thus, ginsenosides may be used alone or in combination with other pharmaceutical agents in new therapeutic strategies for cancer. To date however, there is little systematic summary available for the anticancer effects and therapeutic potential of ginsenosides. Therefore, we have reviewed and discussed all available literature in order to facilitate further research of ginsenosides in this manuscript.

## 1. Introduction

Today, a growing number of people worldwide have cancer, regardless of wealth or social status. According to a WHO report, 18.1 million people worldwide had cancer in 2018, 9.6 million people died of the disease, and these figures are expected to double by 2040 [1].

At present, the clinical management of cancer always involves several conventional modalities, including surgical resection, radiotherapy, immunotherapy, biotherapy, and chemotherapy [2]. Chemotherapy is currently the most commonly used treatment for cancer [3]. Given that it is difficult to determine an appropriate dosage of conventional chemotherapeutic agents, side effects, such as a reduction in bone density and immunosuppression, often result from high doses, while low levels may not produce desired effects [2]. In addition, some chemotherapeutics may cause acquired drug resistance. Thus, as the importance of developing a new agent capable of selectively inducing cancer cell death without threatening normal cells emerges, interest in natural products has been amplified.

Over the last few decades, after the introduction of Western medicine into Eastern countries, traditional medicine has been used in the form of complementary and alternative medicine. Herbs and ingredients of traditional medicine are the focus of active research that aims to incorporate them into standard medical treatments.

Ginseng, *Panax ginseng* C.A. Meyer, has been widely used as a natural tonic in Asian countries, including Korea and China, since ancient times [4]. Among its several active ingredients, ginseng saponins (ginsenosides) are known as the main bioactive agents with various pharmacological activities [5–7]. Many studies have consistently reported that ginsenosides have anticancer effects in several cancer models, even though the exact anticancer mechanism has not been elucidated [8–11]. Therefore, in this review, we summarize and discuss the potential roles of ginsenosides in several cancers.

## 2. Ethnopharmacological Relevance

Ginseng is a plant belonging to the *Panax* genus in the family Araliaceae, and its official scientific name is *Panax ginseng* [12]. The roots of this plant have been used most frequently for traditional therapeutic purposes for the healing and prevention of human diseases [4]. The oldest document that recorded the usage of ginseng, mainly as a tonic to nourish the vitality of a weak body and as supplementary ingredient for other prescriptions, was written around 2000 years ago [13, 14]. Indeed, ginseng's genus name *Panax* means “cure all” in Greek, indicating its potential for treating any illness in traditional medicine [15, 16].

Hence, the pharmacological properties of ginseng have been verified by modern science, and its confirmed medicinal effects include immune response boosting [17], anti-inflammatory [18], hepatoprotective [19], antiobesity [20], antimicrobial [21], cognition enhancement [22], and antioxidant effects [6]. Due to its effectiveness for various health situations, ginseng is a popular choice for health products, dietary supplements, and food and cosmetic products [14]. Also, the root of ginseng is currently used for treating cardiovascular diseases [7], autoimmune diseases [23], Alzheimer's disease [24], stress-induced diseases [25], ocular disease [26], and diabetes [5]. Particularly, many studies have suggested that ginsenosides, the saponin components in ginseng, could suppress proliferation, invasion, and inflammation in several cancer cell lines [10, 27–29]. Correspondingly, several clinical studies have reported that ginseng administration could augment the efficacy of therapeutic drugs in patients with cancer [30, 31]. Oh et al. observed that ginsenosides decreased the viability of breast cancer cells [29], and Kim et al. reported that these compounds blocked the invasion and migration of colon cancer cells [28]. Moreover, Kim et al. [30] and Kim et al. [31] reported that ginseng reduced cancer-related fatigue in colorectal cancer patients with chemotherapy. It is known that cancer-related fatigue severely worsens prognostic outcomes and limits therapeutic options [32]. These results suggest that ginseng has anticancer effects against several cancers and can alleviate the serious side effects associated with cancer treatment.

## 3. Components of Ginseng

**Figure 1:**
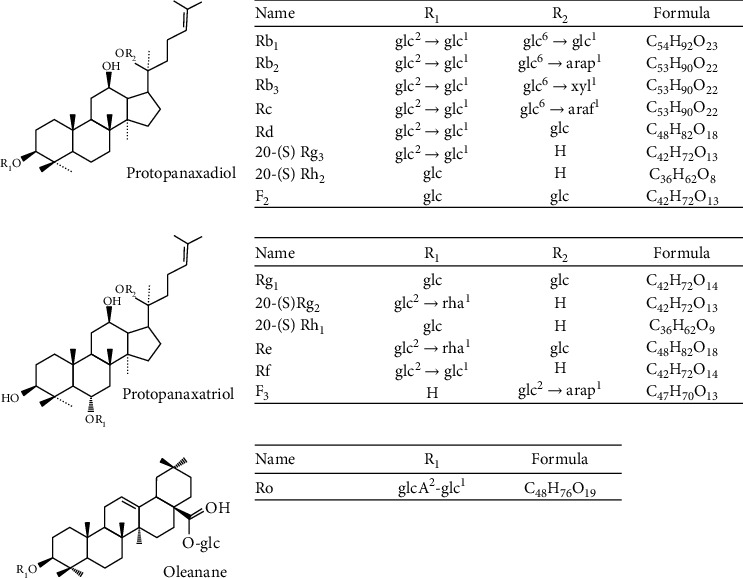
The chemical structure and classification of ginsenosides. Aaraf: *α*-L-arabinofuranosyl; Arap: *α*-L-arabinopyranosyl; Glc: *β*-D- glucopyranaosyl; Rha: *α*-L-rhamnopyranosyl; Xyl: *β*-D-xylopyranosyl.

Chemical composition of ginseng can be mainly divided into two main types: saponin and non-saponin. Most ginseng components are non-saponin and include carbohydrates, nitrogen-containing compounds, fat soluble components, minerals, and vitamins [33, 34]. Carbohydrates in ginseng include polysaccharides, oligosaccharides, sugar, fiber, and pectin, with the largest and active carbohydrates being polysaccharides, comprising 50–60% of ginseng components [33, 35]. The next major components in ginseng are nitrogen-containing compounds—protein, peptides, amino acids, nucleic acids, and alkaloids [36], while fat soluble components consist of fatty acids, essential oils, phytosterol, organic acids, phenolics, and polyacetylenes [37]. However, saponins are the most studied bioactive components of ginseng. Saponins, mostly known as ginsenosides, are derived from 2,3-oxidosqualene. Depending on the enzyme involved, different types of ginsenoside precursors are produced, dammarenediol-II and *β*-amyrin, which are later transformed into tetracyclic dammarane-type and pentacyclic oleanane-type saponins, respectively [38]. Further, dammarane-type saponins are of two different categories, depending on the position of the hydroxyl groups and double bond of their genins [39]. More than 100 ginsenosides have been documented, including 66 protopanaxadiol (PPD), 50 protopanaxatriol (PPT), and 19 oleanane-type ginsenosides [40]. The chemical structures and classifications of ginsenosides are shown in Figure 1.

The diversity of ginsenosides is related to their stereoisomers and constitutional isomers. Some ginsenosides, such as Rg2, Rg3, Rh1, and Rh2, exist in different stereoisomeric forms, 20-(S) and 20-(R), depending on the position of the hydroxyl group in C-20. Other ginsenosides, including Rb2, Rb3, Rc, aglycone, Rg1, and F11, contain different saccharide substituents [41, 42]. However, not all ginsenosides occur naturally in ginseng. Several factors influence the ginsenoside components of ginseng: cultivation method, harvesting age, and preparation treatment [43–45].

In general, wild ginseng is believed to have better pharmacological effects compared to the cultivated one. However, due to increasing demand and excessive gathering of wild ginseng, wild simulated ginseng grown in forests and ginseng cultivated in fields are now mostly used. Forest-grown wild simulated ginseng has more Rb1 and Re content than field cultivated ginseng [46]. Another important factor is the timing of the harvest. Ginsenosides are mostly accumulated in the leaves in young plants (1-2 years old) and amassed in roots in later ages (around 5 years); thus, cultivated ginsengs are mostly harvested around 4–6 years old [47]. Moreover, ginseng harvested in the spring has the most total saponin contents than those of other seasons, and Re contents are higher in late spring and lower in the fall than Rg1 contents [48, 49]. Harvested ginseng can be consumed in their fresh state or after drying. Dried ginseng is commonly referred to as white ginseng, while red ginseng is prepared by steaming the fresh root of ginseng. Steaming changes the chemical composition of ginseng, such that white and red ginseng contain different amounts or varieties of ginsenosides [4]. Naturally occurring ginsenosides can be extracted from harvested fresh ginseng, but most ginsenosides are converted from their original forms by physical, chemical, or biotransformation methods [44]. For instance, major ginsenosides (Rb1, Rb2, Rc, Rd, Re, and Rg1) can be deglycosylated into minor ginsenosides (F2, Rh2, Rg2, Rg3, and CK) [50]. Genetics also plays a role in ginsenoside content, as Korean ginseng (*P. ginseng*) has more major ginsenosides than the Chinese (*P. notoginseng*) or American (*P. quinquefolius*) species [45].

Ginsenosides, important bioactive components in ginseng, have various pharmaceutical activities; however, we will discuss their recently discovered anticancer effects in this review.

## 4. Breast Cancer

Breast cancer is the most common cancer among women and has a high mortality rate when detected at the late stage. However, breast cancer detected early has a lower mortality rate compared to the incidence rate due to the high probability of cure [51, 52]. One of the most studied ginsenosides, Rg3, suppressed breast cancer via several intertwined biological pathways related to cell division and protein synthesis, such as by inhibiting Akt-mediated self-renewal signaling in MCF-7 breast cancer cells [29, 53]. Additionally, Rg3 can inhibit the growth of breast cancer cells by downregulating NOX4 and upregulating KDM5A via altering epigenetic methylation [54]. Similarly, Rh2-modulated epigenetic methylation of immune response-related genes can hinder cancer cells growth [55]. Rd also suppressed metastasis by downregulating miR-18a-mediated Smad2 expression and inhibiting angiogenesis-related vascular endothelial growth factor- (VEGF-) induced Akt activation in cultured or mice xenograft model of MDA MB-231 and 4T1 cells [56, 57]. Similar to the blocking of breast cancer metastasis by halting Akt-activated cell proliferation by Rg3 and Rd, CK ginsenoside repressed Akt1 signaling to promote apoptosis in SKBR3 cells [29, 57, 58]. Moreover, CK, Rg5, and Rk1 suppressed tumor growth in mice xenograft models by inhibiting cyclin D1, phosphatidylinositol-3-kinase (PI3K)/Akt, and reactive oxygen species (ROS)/PI3K/Akt signaling pathway, respectively [59–61]. The activation of the PI3K/Akt pathway, a key regulator of survival, results in the inference of the control of cell growth and survival, ultimately leading to metastatic competence, angiogenesis, and therapy resistance [62].

Furthermore, Rg2, Rg5, and CK ginsenoside derivatives induced autophagy, apoptosis, and cell cycle arrest by regulating p53 expression in MCF-7 breast cancer cells [63–65]. Rh2 combined with PPT had a synergistic effect in delaying cell proliferation in the breast cancer cell line MDA-MB-231 [66]. Interestingly, stereoisomerism also plays a role in the potency of ginsenosides; for example, of the two isomers of Rg3, 20-(R) was more effective in obstructing metastasis (migration and invasion) in MDA-MB-231 breast cancer cells [67]. Taken together, ginsenosides exhibit their anticancer effects by inhibiting differentiation, proliferation, and metastasis and induce autophagy, apoptosis, and cell cycle arrest by altering pathways related to carcinogenesis in breast cancer cell lines.

## 5. Brain Cancer

Brain cancer is a tumor located in the brain or central nervous system. The incidence rate of primary brain cancer is relatively low; however, patients with Parkinson's disease have a higher risk of the disease [68, 69]. About two-thirds of all brain cancers in adults are glioblastoma, the most malignant type of brain cancer with a high mortality rate, in which patients die within 1-2 years of diagnosis [70]. Poorer prognosis of glioblastoma is correlated with the activation of various signaling pathways including the Wnt/*β*-catenin signaling pathway [32, 71]. The binding of the Wnt ligand to the Frizzled receptor stabilizes the transcriptional regulator *β*-catenin, which translocates from the cytosol to the nucleus, where it binds to the transcription factor T-cell factor/lymphoid enhancer-binding factor (TCF/LEF) to promote the expression of its downstream genes, such as TCF1, LEF1, cMYC, and CCND1 [32]. Recent studies have shown that Rh2 and Rg3 inhibited migration, proliferation, invasion, and cancer-related inflammation via downregulating Wnt/*β*-catenin signaling in glioblastoma [32, 72].

Rg3 also improved the efficiency of temozolomide, a drug for treating glioblastoma, in temozolomide-resistant glioblastoma [73, 74]. Additionally, Rg3, Rb1, and F1 ameliorated senescence, which is one of the side effects of chemotherapy treatment in glioblastoma, and had neuroprotective properties against brain-related diseases [75–77]. Rh2 reduced proliferation, apoptosis, and metastasis via downregulating miR-31 to suppress Wnt/*β*-catenin signaling and Akt activities, thereby blocking the activation of matrix metalloproteinase (MMP)13, a major degradation enzyme in Daoy medulloblastoma and glioblastoma cells [72, 78]. Similarly, CK induced cell arrest and apoptosis by repressing the PI3K/Akt/mTOR pathway in glioblastoma cell lines, U87MG and U373MG [79]. Therefore, ginsenosides inhibit proliferation and induce apoptosis, possibly by blocking signaling pathways related to the deterioration of glioblastoma.

## 6. Liver Cancer

Hepatocellular carcinoma (HCC) is the most common primary liver cancer; it accounts for 80% of the total cases and is one of the leading causes of cancer-related deaths [80]. Primary risk factors of liver cancer are cirrhosis, hepatitis viral infection, and alcohol consumption [80, 81]. PPD reduced viability and induced apoptosis in HepG2 cells by suppressing the PI3K/Akt pathway [82]. One of the PPDs Rg3 showed antimetastatic effects by upregulating Rho GTPase activating protein 9 (ARHGAP9) and downregulating NHE1 expression in vivo and in vitro [83, 84]. The overexpression of ARHGAP9, a member of the RhoGAP family, inhibited HCC cell proliferation, migration, invasion, and metastases [85]. Moreover, Rh2 ginsenoside inhibited HCC cell proliferation and growth by modulating *β*-catenin signaling and epidermal growth factor receptor (EGFR) signaling. Also, the potency of Rh2 ginsenosides can be increased by upregulating the expression of miR-491 and miR-146a-5p, which play important roles in the development and progress of HCC [86–88]. Compared to the isomer 20-(R), 20-(S) Rh2 reduced cancer cell growth by inactivating Annexin A2; however, there were no differences in effectiveness between the stereoisomers, given that both isomers induced cell apoptosis by downregulating Bcl-2 mRNA in a mouse model of hepatoma [89, 90]. Interestingly, Rg1 not only sensitized hepatoblastoma to DNA-damaging agents but also protected D-gal-induced liver injury in mice by reducing oxidative stress and DNA damage [91, 92]. CK can induce apoptosis by inhibiting the phosphorylated signal transducer and activator of transcription (STAT) 3 in human HCC xenografted mice models [93]. However, its synthetic mono-octyl ester form had a greater inhibitory effect in murine hepatic carcinoma cell line (H22)-bearing mice than CK [94].

Ginsenosides also enhanced the efficiency of existing drugs for treating liver cancer. For instance, Rg3 combined with sorafenib or oxaliplatin had synergetic effects on inhibiting proliferation, promoting apoptosis, and decreasing tumor volume via regulating the PTEN/Akt signaling pathway or proliferation cell nuclear antigen (PCNA)/cyclin D1 expression, which are key regulators of many cellular processes [95, 96]. Moreover, Rh2 combined with regorafenib suppressed HCC cell growth by upregulating caspase-3 expression and modulating the survivin gene, a member of the apoptosis-inhibiting gene family [97]. Rg3 alleviated transcatheter arterial chemoembolization syndromes and increased survivability in patients with advanced hepatocellular carcinoma [98]. Altogether, ginsenosides reduce cell viability, inhibit tumor growth, and promote apoptosis in HCC independently or in combination with existing drugs.

## 7. Gastric Cancer

Gastric cancer is globally ranked third among the causes of cancer-related death, and the majority of cases are discovered at the late stage, with almost half of the recorded cases from East Asian countries, such as China, Japan, and South Korea [99, 100]. A report showed that PPD including 25-hydroxyprotopanaxadiol (25-OH-PPD) and 25-methoxylprotopanaxadiol (25-OCH3-PPD) had potent anti-tumor properties, such as cell growth inhibition and apoptosis induction in gastric cancer cells lines [101]. Furthermore, 2-Pyrazine-PPD and 4-XL-PPD, the derivatives of 25-OH-PPD, induced apoptosis by producing ROS [102, 103]. Several studies reported that Rg3 ginsenoside repressed the metastasis of gastric cancer by inhibiting hypoxia-induced factor-1*α* (HIF-1*α*) and VEGF expression [104, 105]. In addition, Rg3 had a preventive effect against the risk factors of gastric cancer, gastric precancerous lesions, and *Helicobacter pylori* [106, 107]. Rg5 blocked cell growth by inducing G2/M phase cell cycle arrest, apoptosis, and autophagy in xenografted models [108]. Also, Rh2 and Rd induced G0/G1 phase cell cycle arrest and apoptosis by regulating the Bax/Bcl-2 ratio, which are key proteins associated with apoptosis [109, 110]. In summary, ginsenosides not only restrict cell viability but also induce apoptosis, cell cycle arrest, and autophagy in gastric cancer cell lines and mice models via regulating the expression of several genes.

## 8. Lung Cancer

Lung cancer, which can be divided into two classes, small-cell and non-small-cell lung carcinomas (NSCLC), is mostly caused by smoking and is the leading cause of cancer-associated mortality worldwide [111]. The most diagnosed lung cancer is NSCLC, which accounts for about 85% of lung cancer incidence [111, 112]. Consequently, most ginsenoside studies on lung cancer have been focused on NSCLC. A recent study reported that C_3_C_12_ PPD, a newly discovered ginsenoside, inhibited cell growth, proliferation, migration, and tube formation of NSCLC in vivo and in vitro by inhibiting several signaling pathways, such as Raf/MEK/ERK, AKT/mTOR, and AKT/GSK-3*β*/*β*-catenin [113]. Furthermore, CK suppressed cell viability by downregulating HIF-1*α* mediated glucose metabolism and increased autophagy-mediated apoptosis via AMPK-mTOR and JNK signaling pathways in NSCLC [114]. Rg3 inhibited epithelial-mesenchymal transition (EMT) and tumor growth by suppressing FUT4-mediated EGFR inactivation and the PI3K/Akt signaling pathway, as well as by promoting vaccinia-related kinase (VRK)1 expression [115–117]. Rk3 abrogated NSCLC xenograft tumor growth by causing cell cycle arrest at the G1 phase and apoptosis [118]. Similarly, Rg18 mediated G1 phase cell cycle arrest in A549 human NSCLC cells [119]. Rh2 converted tumor-associated macrophages from the M2 to M1 subset to prevent migration of lung cancer cells and repressed migration in the hypoxic environment via upregulating miR-491 expression [120, 121]. Additionally, Rh2 restrained NSCLC cell proliferation by inducing ROS-mediated cell apoptosis [122].

Ginsenosides have been known to improve the efficiency of currently used treatments against lung cancer. Rg3 improved sensitivity and suppressed resistance to several drugs, such as icotinib, gefitinib, cisplatin, gemcitabine, and osimertinib [123–128]. Several studies observed that Rk1 and Rg3 restricted programmed death ligand 1 (PD-L1) expression, Rg3 improved the efficacy of the epidermal growth factor receptor-tyrosine kinase inhibitor (EGFR-TKI), and Rd downregulated the NRF2 pathway to decrease acquired chemoresistance [128–131]. Moreover, several researchers have demonstrated that the anticancer effect and delivery of ginsenosides, such as Rh1, Rh2, and CK, can be further improved by conjugating them to polyethylene glycol (PEG), liposomes, and D-*α*-tocopherol polyethylene glycol 1000 succinate (TPGS) [132–136]. Overall, ginsenosides exhibit their anticancer and antimetastatic properties in lung cancer cell lines and mice models via modulating signaling pathways and can promote the efficiency of current drugs.

## 9. Conclusion

Many in vivo and in vitro studies demonstrated that ginsenosides inhibit the growth and spread of several cancers by altering various signaling pathways and gene expression (Table 1). Although there have been a few clinic trials, most recent anticancer studies of ginsenosides were performed in cancer cell lines and mice xenograft models, with PPD ginsenosides being the most studied. Specifically, the ginsenosides Rg3 and Rh2 were the main focus of many studies and have been shown to have strong anticancer effects. Ginsenosides display their anticancer and antimetastatic properties not only via restricting cancer cell proliferation, viability, invasion, and migration but also by promoting apoptosis, cell cycle arrest, and autophagy. Moreover, the synergistic effect of ginsenosides with existing cancer therapies has been confirmed. Acquired chemoresistance can be reversed or suppressed by ginsenosides, improving sensitivity to drugs in patients treated with chemotherapy. Taken together, ginsenosides have been proven to be effective anticancer agents through many studies; however, more clinical trials are needed to determine whether ginsenosides can be used as the main ingredients for new drugs or as supplements to existing cancer treatments.

## Figures and Tables

**Table 1:** Anticancer effects of some ginsenosides.

Ginsenoside	Function	Cancer	Reference
Rd	↓proliferation, migration, invasion, metastasis, angiogenesis, colony formation ↑ apoptosis, cell cycle arrest, sensitivity to cisplatin	Breast Gastric Lung	[56, 57], [109], [130]

CK	↓ cell viability, tumor growth, invasion, migration, colony formation, metastasis, self-renewal capacity, self-renewal ↑ cell cycle arrest, apoptosis, autophagy	Breast Brain Liver Lung	[58, 60] [79] [93, 94] [114, 135, 136]

Rg1	↓ tumor growth, ↑ hepatoprotective	Liver	[91, 92]

Rg2	↑ autophagy	Breast	[64]

Rg3	↓ cell viability, self-renewal capacity, proliferation, spheroid formation ability, angiogenesis, metastasis, migration, invasion, EMT ↑ apoptosis, autophagy, cell cycle arrest ↑ sensitivity to sorafenib, osimertinib, oxaliplatin, cisplatin, temozolomide, icotinib, gefitinib	Breast Brain Liver Gastric Lung	[29, 53, 54] [32, 73–75] [83, 84, 95, 96] [104, 105] [115–117, 123–128]

Rg5	↓ proliferation, tumor growth ↑ cell cycle arrest, apoptosis,	Breast Gastric	[59, 62] [108]

Rg18	↓ proliferation ↑ cell cycle arrest	Lung	[119]

Rh1	↓ viability	Lung	[134]

Rh2	↓ tumor growth, proliferation, invasion, angiogenesis, colony formation, migration, metastasis ↑ apoptosis, autophagy, cell cycle arrest	Breast Brain Liver Gastric Lung	[66] [72, 78] [86–90, 97] [109] [120–122]

Rk1	↓ tumor growth, proliferation, colony formation, ↑ apoptosis, cell cycle arrest	Breast Lung	[61] [131]

Rk3	↓viability, proliferation and colony formation, angiogenesis ↑ cell cycle arrest, apoptosis	Lung	[118]

## Data Availability

No data were used in the study.
